# Randomised controlled single-blind study of conventional versus depot mydriatic drug delivery prior to cataract surgery

**DOI:** 10.1186/1471-2415-6-36

**Published:** 2006-11-27

**Authors:** Vincent Dubois, Nadia Wittles, Meon Lamont, Simon Madge, Jon Luck

**Affiliations:** 1 Eye Unit, Royal United Hospital, Combe Park, Bath, B&NES, BA1 3NG, UK

## Abstract

**Background:**

A prerequisite for safe cataract surgery is an adequately dilated pupil. The authors conducted a trial to assess the efficacy (in terms of pupil diameter) of a depot method of pre-operative pupil dilatation, as compared with repeated instillations of drops (which is time-consuming for the nursing staff and uncomfortable for the patient).

**Methods:**

A prospective randomised masked trial was conducted comprising 130 patients with no significant ocular history undergoing elective clear corneal phacoemulsification. 65 patients had mydriatic drops (Tropicamide 1%, Phenylephrine 2.5%, Diclofenac sodium 0.1%) instilled prior to surgery, 65 had a wick soaked in the same drop mixture placed in the inferior fornix. Horizontal pupil diameters were recorded on a millimetre scale immediately prior to surgery.

**Results:**

There was no significant difference in pupil size between the two groups (p = 0.255, Student's t-test).

**Conclusion:**

There was no significant difference between the mydriasis obtained with the depot system compared with conventional drop application. Use of a depot mydriatic delivery system appears to be a safe and efficient method of drug delivery.

****Trial Registration**:**

International Standard Randomised Controlled Trial Number Register ISRCTN78047760

## Background

A prerequisite for safe cataract surgery is an adequately dilated pupil. Various methods of mydriatic drug delivery have been studied, including sprays and depots, with the common goal of obtaining maximal dilatation of the pupil efficiently and comfortably [1-3]. Some studies have also looked at combining the dilating agents into single preparations for ease of use [1,3-5].

A previous study of corneal anaesthesia produced by depot drug application showed that this produced longer corneal anaesthesia compared with drops, despite there being a smaller drug concentration overall [5]. This increased 'bioavailability' is the common principle behind all forms of depot mydriatic delivery, and formed the basis for our study.

A recent uncontrolled study [6] showed that a change in practice to using a pre-soaked wick had acceptable results with respect to pupil dilatation. Our study was carried out in a controlled, prospective, randomised, and masked fashion in an attempt to provide sound evidence of this suggested effect.

## Methods

Ethics committee approval was obtained prior to commencement of the study. Patients who were scheduled for routine cataract surgery were selected for participation in the study. Exclusion criteria were a history of diabetes, uveitis, any pupillary abnormalities, pseudoexfoliation, use of miotics (but not other glaucoma medications), and very dark irides; all of these might prevent reliable assessment of pupillary dilatation with the naked eye or may be a cause for inadequate/irregular pupil dilatation.

Fully informed patient consent was obtained prior to participation. 130 patients were enrolled into the study, each of whom was about to undergo unilateral cataract extraction via small incision clear corneal phacoemulsification. This number was obtained from a power calculation, looking for a medium effect size (0.5), with p = 0.05, aiming for α = 0.8, allowing for a drop-out rate. Patients were randomised into two groups of 65 cases, and 65 controls. An envelope was opened if the patient signed the consent form, which contained either the word WICK (for the cases), or DROPS (for the controls), and the patient was assigned to their respective group.

The DROPS group received a statim dose of Diclofenac sodium 0.1%, and then alternate Tropicamide 1% and Phenylephrine 2.5% at 15-minute intervals in the pre-operative hour (i.e. four of each irrespective of observed pupil size). This is the local standard practice.

The WICK group received a statim dose of proparacaine followed by insertion of a mydriatic-soaked wick (BD visidrain™ eye fluid wick, cut into 1 cm strips) into the lower fornix. The wick used in the WICK group had been soaked in an equal part mixture of the above drops. No further topical medications were applied to the WICK group, and it was ensured at the time of placement that the wick was completely obscured from view.

In the local Eye Unit, patients for cataract surgery all arrive at the same time, 1.0 hours prior to start time. All the patients receive dilating drops (or wicks) at the same time. Their subsequent pupil measurements occur between 1.0 and 4.0 hours from instigation of mydriasis. Upon arrival in the anaesthetic room, all patients had measurement of horizontal pupil diameter with a pair of callipers to within half a millimetre by a masked observer (who was the doctor administering the local anaesthetic). The same callipers were used throughout the study period. Callipers are precision instruments, easily obtainable and easy to use, which is why this method was used for assessment of pupil diameter. Subsequently, the doctor checked for the presence or absence of a wick in the inferior fornix and removed it with forceps if in situ. Pupil diameter and presence/absence of a wick were both recorded on the data sheet. Data on intra-operative and post-operative pupil diameters were not collected.

## Results

**Figure 1:**
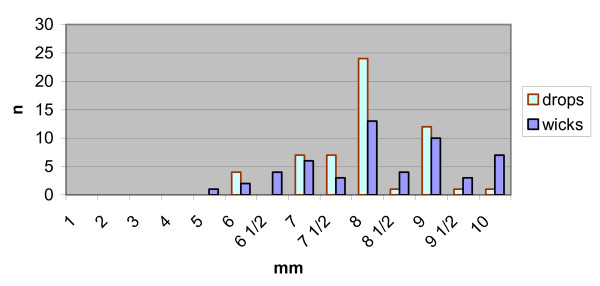
Graph of study results Pupil diameters.

There was no significant difference between the observed pupil sizes in the two groups of patients (Student's t-test: p = 0.255, Table 1 & Figure 1).

**Table 1:** Pupil diameters (mm)

	Mean	Median	Standard Deviation	No. of Patients (n)
Wicks	8.198	8	1.213	53
Drops	7.968	8	0.877	57

Statistical value p = 0.255

One wick was inadvertently left in situ by the doctor administering the local anaesthetic, and was subsequently removed by the surgeon in the operating theatre. Having been wrongly noted to have 'no wick' in situ, this was subsequently corrected.

No adverse events were experienced in either group, and surgery was uncomplicated in all cases. Importantly, there were no adverse incidents secondary to inadequate pupil dilatation in either group. No patient was unable to tolerate the wick. One wick fell out shortly after insertion, so that patient was excluded from the study. A small dropout rate occurred secondary to improper or incomplete filling in of forms, hence there are not 65 responders in each group, as shown by 'n' in the tables.

## Discussion

In this single-masked, randomised controlled trial we have demonstrated that pupil dilatation using a depot method of mydriatic drug delivery is no different to the local practice in achieving a pre-operative pupil diameter which allowed safe cataract surgery during the study period.

Previous studies [1-3] have examined novel ways of administering mydriatics prior to cataract surgery. Siderov et al [7] showed a small, statistically significant, but not clinically significant difference in pupil sizes when subjects received tropicamide alone, compared with tropicamide following administration of proparacaine (in favour of the proparacaine group). This was only true for patients with light-coloured irides. Mechanisms suggested for this difference were disruption of the corneal epithelium, and reduced reflex tearing through increased comfort. However, Siderov et al found only a paucity of data supporting the use of proparacaine in conjunction with tropicamide alone. There is significant data supporting the facilitation which proparacaine offers in the pupillary dilatation with phenylephrine. [8-10] These factors would have resulted in increased bioavailability in our study, however we were unable to demonstrate any such effect in our WICK group.

There was a large variability in the delay between instigation of mydriasis and measurement of pupil diameters. This is due to the logistical nature of running a busy cataract day surgery unit in a District General Hospital in the UK. However, given that patients in both groups were exposed to this random variability, and given the substantial numbers of participants in each group, then the effect of this confounding factor would be greatly reduced.

Apt and Henrick did find a consistent and statistically significant increase in pupil sizes [5] depending on whether proparacaine was used prior to instillation of various mydriatic preparations. Our WICK group may or may not therefore have had an enhanced mydriatic effect following the prior administration of proparacaine, meaning that without proparacaine the WICK method may not actually be as effective as standard drop instillation as described above.

Ong-Tone showed that satisfactory pupillary dilatation was achieved when mydriatic-soaked wicks were used to dilate pupils pre-operatively, as compared with standard drop instillation [6]. The main outcome measure was that pupils were noted to be adequately or inadequately dilated. The study found no statistical difference between the two groups. There was no mention of blinding or randomisation, nor were there any numerical data on the sizes of the pupils achieved with either method. There was also no definition of what constituted an adequately or inadequately dilated pupil. Consequently, we found it difficult to draw sound conclusions from this study. We deliberately designed our study to be conducted in a prospective, randomised, single-blind case-controlled manner, in order to exclude bias.

There is also the unmeasured effect on nursing resource and the economics of surgery. We noted anecdotally during the study period that the nursing staff found application of the wick a simple, efficient, one-off event, and it freed them up for other duties. Based on the costs of the drops and other materials used at the time of the study, we calculated a saving of approximately three pounds sterling per session, based on five cataract patients per list. When applied to the significant volume of cataract surgery carried out in Europe and abroad, this represents a not inconsiderable saving.

## Conclusion

Use of a wick pre-soaked in standard mydriatic and non-steroidal anti-inflammatory drugs appears to be a safe and effective method of pupillary dilatation prior to routine cataract surgery which is well tolerated by patients in the presence of proparacaine. Additional benefits may include a saving both in financial terms and in the nursing resource required in a busy cataract unit.

## Competing interests

The author(s) declare that they have no competing interests.

## Authors' contributions

VD designed the study, wrote the protocols, helped recruit the patients, and wrote the paper.

NW helped recruit the patients, performed statistical analysis, and co-wrote the paper.

ML and SM assisted in study design, and helped recruit the patients.

JL oversaw the trial and gave final approval for the manuscript.

All authors read and approved the final manuscript.

## Pre-publication history

The pre-publication history for this paper can be accessed here:


